# SYMPTOM BURDEN AND RECURRENT FALLS IN OLDER CANCER SURVIVORS: A LONGITUDINAL POPULATION-BASED STUDY

**DOI:** 10.1093/geroni/igae098.3369

**Published:** 2024-12-31

**Authors:** Myeongjin Bae, Kushang Patel, Nancy Gell

**Affiliations:** University of Vermont, Burlington, Vermont, United States; University of Washington, Seattle, Washington, United States; University of Vermont, Burlington, Vermont, United States

## Abstract

Older adults with a cancer history may be at greater fall risk as activity-limiting symptoms from cancer and cancer treatment often persist, even after treatment has ended. Therefore, we aimed to prospectively determine the effects of pain, fatigue, and sleep difficulty on fall risk in older adults with a cancer history. Data from the National Health and Aging Trends Study Rounds 1-5 (2011-2015) were analyzed. The study sample (n=1443) was limited to community-dwelling Medicare beneficiaries who self-reported any history of cancer, other than skin cancer only. Symptoms were assessed through questions regarding pain, fatigue, and sleep difficulty. The main outcome measure was the cumulative incidence of recurrent (≥2) falls in at least one year from 2011. All analyses were weighted and accounted for complex sampling design. Incidence of recurrent falls was significantly higher among those with pain, fatigue, and sleep difficulty. In adjusted models, compared with those with no symptoms, the adjusted risk ratios for recurrent falls were 1.80 (95% confidence interval [CI] = 1.31–2.48), 2.10 (95% CI = 1.61–2.75), and 1.66 (95% CI = 1.25–2.20) for older cancer survivors with pain, fatigue, and sleep difficulty, respectively. For those with all three symptoms, the adjusted risk ratio for recurrent falls was 4.58 (95% CI = 2.82–7.42), compared to those with no symptoms. This study revealed the significant impact of aging and cancer-related symptoms on fall risk in older adults with a cancer history. Reducing symptom burden is needed to mitigate fall risk in this population.

